# Specialist Palliative Care and Dementia: Staff Challenges and Learning Needs

**DOI:** 10.1177/08258597231180966

**Published:** 2023-06-21

**Authors:** S.J. Currie, C. Curtin, S. Timmons

**Affiliations:** 1 8795 University College Cork, Cork, Ireland

**Keywords:** specialist palliative care, dementia, challenges, learning needs, education

## Abstract

**Objective:** This study explored the perspectives of specialist palliative care (SPC) teams in Ireland, in relation to personal learning needs and education regarding dementia care. **Methods:** This mixed-methods study involved a survey and focus group. SPC staff were recruited through a professional palliative care society and via hospices in 4 regions. Survey items included challenges in clinical care, personal learning needs, and preferred modes of educational delivery. Quantitative data analysis was descriptive; open-answer survey questions and the focus group transcript underwent thematic analysis. **Results:** In total, 76 staff completed surveys and rated the following as most challenging: timely access to community agency and specialist support; and managing the needs of people with dementia (PwD). Respondents volunteered additional challenges around the timing/duration of SPC involvement, prognostication, and inadequate knowledge of local services. Staff ranked learning needs as highest in: nonpharmacological management of noncognitive and cognitive symptoms; differentiation of dementia subtypes; and pharmacological management of cognitive symptoms. The focus group (n = 4) gave deeper perspectives on these topics. Overall, 79.2% of staff preferred formal presentations by dementia-care specialists and 76.6% preferred e-learning. **Conclusion:** Several dementia-care challenges and learning needs are identified by SPC staff, as above. These can inform the design and delivery of tailored education programs for SPC staff. There is also a need for closer working between dementia services and SPC services to provide integrated, holistic care for PwD. One aspect of achieving this is greater awareness of local dementia-care services among SPC staff, and vice versa.

## Introduction

Dementia is a global concern and a public health priority.^1^ The World Health Organization estimates that approximately 55 million people are living with dementia and nearly 10 million new cases are reported annually.^1^ The total number of people with dementia (PwD) will likely reach 152 million by 2050.^1^ Dementia is not a normal aspect of ageing.^1^ It is a devastating, life-limiting, and incurable neurodegenerative condition that leads to impaired memory, communication, problem-solving, and ultimately, the ability to perform activities of daily living.^1^

Dementia warrants high-quality palliative care.^2,3^ Several international reviews have highlighted the importance of palliative care in neurodegenerative conditions with no curative treatments, including dementia.^4,5^ Notably, the Irish National Dementia Strategy emphasized palliative care as a key component of dementia care.^6^ Palliative care is an approach that focuses on improving quality of life through the prevention and relief of suffering, beginning at the time of diagnosis and continuing to end-of-life care.^7^ It is well-established that PwD and their caregivers have palliative care needs similar to people with cancer.^2,8,9^ PwD require assessment and management of symptoms such as pain, dyspnea, anorexia, swallowing difficulties, fear and agitation, seizures, pressure ulcers and communication difficulties.^2,3,10^ However, evidence shows that many PwD are not assessed to determine their palliative care needs.^2^ Compared to people with cancer, PwD receive suboptimal palliative and end-of-life care, with increased hospitalization, inadequate pain control, and less specialist palliative care (SPC) involvement.^11,12^

The provision of palliative care for PwD is a new area of clinical practice for many healthcare professionals.^2,8^ In 2014, the European Association for Palliative Care (EAPC) defined optimal palliative care for PwD.^13^ Consensus was reached on the following domains: person-centered care; communication and shared decision making; optimal treatment of symptoms and providing comfort; setting care goals and advance planning; continuity of care; psychosocial and spiritual support; family care and involvement; education of the healthcare team; and societal and ethical issues.^13^ Notably, the authors concluded that SPC teams may need additional dementia-care specialist support in navigating behavioral symptoms, and advocated for the entire SPC team having adequate skills to enable a palliative care approach to PwD.^13^

Many studies have reported related themes of education, ongoing learning, and practice development, as important in the delivery of optimal palliative care for PwD,^11,14–23^ particularly staff training and education in dementia care and in palliative care.^16,19^ In Ireland, a joint Irish Hospice Foundation and Alzheimer Society of Ireland report found limited awareness, knowledge, and skill-base across disciplines and services, in relation to palliative care for PwD.^24^ Limited research exists exploring the perspectives and experiences of SPC teams caring for PwD. A UK-based study explored physicians’ perspectives around pain management for PwD nearing end-of-life, and included physicians in primary care, dementia, and SPC services. The key areas for further training included pharmacology, pharmacotherapeutics, distinguishing between pain and nonpain-related behavioral and psychological symptoms of dementia (BPSD), and managing pain in the presence of BPSD.^14^ In this study, a hospice-based consultant suggested that physician-to-physician mentoring would help address some of these issues.^14^

To date, to the authors’ knowledge, no study has explored the dementia-care learning needs of SPC teams, despite their key role in a comprehensive approach to a PwD with complex palliative care needs requiring specialist input. We anticipated that SPC teams, although expert in palliative care, might have challenges in providing palliative care to a PwD, and might have unique education needs. The aim of this research was thus to explore and assess SPC teams’ dementia-care challenges, self-identified learning needs, and preferred modes of educational delivery. This will help to inform the design and delivery of tailored education programs for SPC, with the ultimate goal of addressing the poorly met need for palliative care among PwD.

## Methods

### Study Design

This was a mixed-methods study involving a survey (quantitative and qualitative data) and focus group, the former to quantify the problem and the latter providing richer insight into participants’ perspectives.^25^ We used a triangulation approach,^26^ wherein a conventional “convergence model” was employed, with qualitative and quantitative data separately analyzed and results subsequently converged.

### Setting, Participants, and Recruitment

The inclusion and exclusion criteria for selecting participants for the survey and the focus groups were the same. The target study population was SPC doctors, nurses, and health and social care professionals (HSCPs) with some experience of providing clinical care for PwD, who were geographically located and working within Ireland. Participants were recruited to participate in the survey indirectly by email through the Irish Palliative Medicine Consultants’ Association (IPMCA), which has national coverage. In addition, personal emails were sent to SPC consultants in 5 geographically spread hospices (Dublin, Waterford, Limerick, Cork), to act as study champions. All survey recipients were invited to disseminate the survey to their colleagues to maximize the survey reach.

With regard to the focus group recruitment, the final page of the survey informed participants that focus groups were also going to be conducted, and the author's contact email was provided such that participants who were interested in focus group participation could contact the author for further information. In addition, the IPMCA and study champions outlined above sent an email to SPC staff within the same 5 hospices across Ireland containing information and an invitation to participate in the focus groups.

### Data Collection

Data collection was completed in a parallel manner, between September 1, 2021 and January 28, 2022. An online survey was created by minimally adapting an existing Canadian survey that explored the dementia learning needs of primary care clinicians.^27^ This was chosen due to its applicability for a nondementia service, and the absence of a tool specific to SPC staff. With the author's permission and in collaboration with an expert in dementia palliative care, the survey was adapted to improve its suitability and relevance for SPC staff (see Appendix 1). The online survey was created using Google Forms and piloted with a local geriatrician to ensure it was easily understandable by clinical staff.

The survey collected data on staff demographics, challenges, learning needs, and preferred modes of educational delivery. The first 2 sections, which concerned clinical care challenges and learning needs, respectively, used a 5-point Likert scale, from 1 (not at all challenging) to 5 (very challenging). The third question asked participants to select their preferred modes of educational delivery, by checking all items that applied. Each section invited participants to write additional free text comments.

Following survey administration, a parallel online focus group was conducted, on Microsoft Teams, to further explore participants’ perspectives. It was not possible to recruit other focus groups due to significant clinician work pressures at the time. The author underwent training around focus group facilitation with an experienced researcher in qualitative methodology. A topic guide was created by the author team through review of qualitative research methodology (see Appendix 2).

### Data Analysis

An integrated approach to data analysis was used. Descriptive statistical analysis was completed on survey data using Brown-Forsythe and Welch analysis of variance (ANOVA) tests, with GraphPad Prism version 9.0, to examine the statistical significance of differences between survey item responses. Combined thematic analysis explored open-answer survey questions and the focus group transcript, wherein initial coding was completed by SC, with challenge and agreement on final codes with Suzanne Timmons. The Braun and Clarke’s^28^ 6-phase process of thematic analysis was employed to guide the process of data analysis concerning the focus groups and the open-answer survey questions. The flexible nature of the authors’ strategy facilitated the consideration of individual datasets and was compatible with a pragmatic, theoretical approach underpinning the mixed-methods research study.

## Results

### Participant Characteristics

In total, 76 SPC staff completed the survey; Table 1 illustrates their demographics. Due to the inclusion of snowball-type sampling, it is not possible to know the number that received the survey, and hence the response rate.

**Table 1.** Participant Demographics.

	Count	Percentage
Sex		
Female	69	90.8
Male	5	6.6
Prefer not to say	2	2.6
Age		
20-34 years old	13	17.1
35-49 years old	43	56.6
50+ years old	20	26.3
Discipline		
Doctor	20	26.3
Nurse	44	57.9
Physiotherapist	6	7.9
Occupational therapist	5	6.6
Social worker	1	1.3
Years in practice		
<5 years	2	2.6
6-15 years	26	34.2
>15 years	48	63.2
General location in Ireland		
Dublin city or county	26	34.2
East/North East/Midlands	17	22.4
South/South East/South West	22	28.9
West/North West	11	14.5
Settings of practice included		
Community	52	68.4
Inpatient hospice	29	38.2
Inpatient hospital (acute)	19	25.0
Other	6	7.9

Most respondents indicated that they worked in the community (n = 52, 68.4%) and/or in hospices (n = 29, 38.2%), with some working in acute care settings, while “other” settings included outpatient/day services and nursing homes. The focus group comprised 4 SPC doctors from 3 different sites; all were highly experienced in SPC service delivery.

## Survey Results

### Challenges in Providing Clinical Care for PwD

**Figure 1.**
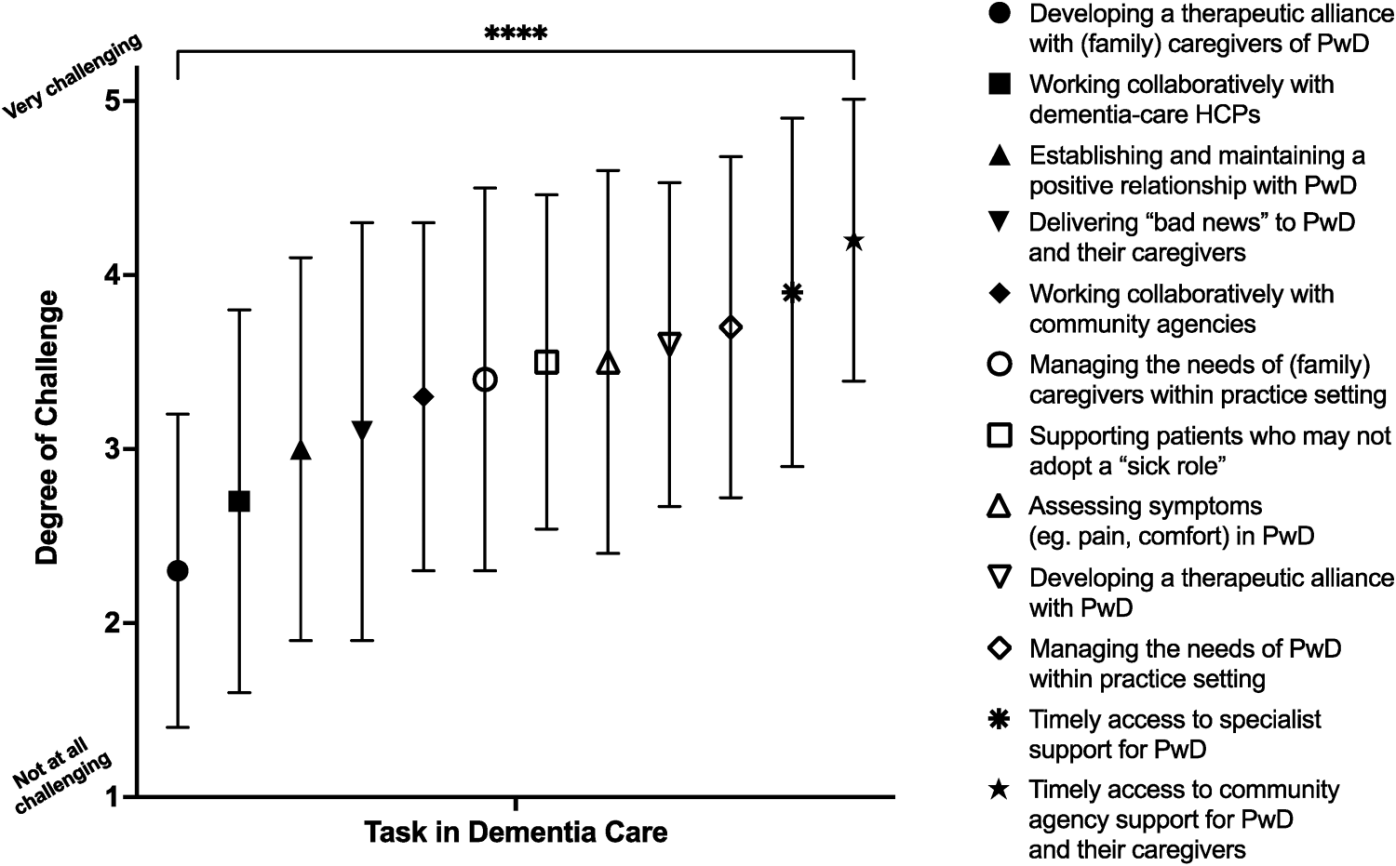
Challenges to providing clinical care for PwD; mean (symbol) and standard deviation (line bars) are shown. Abbreviation: PwD, persons with dementia.

The first survey section related to challenges in providing clinical care for PwD. Figure 1 demonstrates the wide range of degree of challenge between areas of care (*P* < .0001). The least challenging tasks would likely be an intrinsic part of providing SPC for any patient; for example, developing a therapeutic alliance with family members/caregivers (mean 2.3; standard deviation [SD] [0.90]) and delivering “bad news” to patients and caregivers (3.1 [1.2]). SPC staff were most challenged in managing the needs of PwD within the practice setting (3.7 [0.98]), and accessing dementia-care specialist support (3.9 [1.0]) and community agency support in a timely manner (4.2 [0.81]). This latter item had a narrow variance, indicating at least some degree of challenge for all respondents. There was no difference in these identified challenges between doctors (n = 20) and nurses (n = 44), however HSCPs (n = 12) ranked the following as most challenging: supporting patients who may not adopt a “sick role,” and assessing symptoms in PwD.

In the focus group, participants reinforced the strong beneficial role for SPC input for a PwD, and felt that SPC services could particularly support: advance care planning, symptom control, end-of-life care, out of hours access, and emotional support provision. However, limitations in their skills for certain symptoms, where these were felt to be more unique to dementia, was evident throughout the focus group, such as:

*I think there are 3 particular areas that we probably have a role in. One is advanced care planning (…). The second would be around symptom control (…). The third would be end-of-life care where we can come in with good skills around managing someone's death (…). Those are 3 areas where we can borrow skills from other areas of work that we do well. We are not as skilled at managing agitation (…)—*FG Doctor 2.

The focus group discussion included a need to consider when and for how long to be involved in the care of PwD:

*We should not be trying to displace the geriatricians (…). In fact, there is evidence that you get worse outcomes by comprehensively taking over management of people with advanced dementia (…)—*FG Doctor 2.

This issue of optimum timing and duration of input also arose as open-answer responses to the “challenges of care” survey section:

*In palliative care we tend to meet people living with dementia in their final months or days. Many referrals are made to out rule pain as a cause of disruptive or challenging changes in behaviour (…) Often on a first assessment it's very difficult to evaluate pain in a moment in time (…)*—Survey Participant (Nurse).

Prognostication and the prolonged lifespan of some people with dementia was a challenge to SPC services as evident in the following quotations:

*I think one of the big issues is that people with dementia can live quite a long time actually. And prognostication is very difficult, so you’re working against a very obscure prognostic backdrop. That makes judging the trajectory difficult and knowing when to come in and come out difficult—*FG Doctor 2.

*… people with dementia are living longer and the trajectory of the illness can sometimes be over maybe 10 or 15 years (…) I just wonder fundamentally should we be stepping in at that earlier stage at all because it is very hard to step out again.—*FG Doctor 3.

Prognostication, as a learning need, had also arisen in one open-answer survey response:

Would like to learn more about prognosis measurement—Survey Participant (Nurse).

A lack of knowledge surrounding local dementia-care services was highlighted by survey participants’ open text responses, and also in the focus group discussion. The following quote is typical of many participants; here, a doctor indicates that a lack of knowledge regarding the local services of other specialties is an issue experienced across health care professionals (HCPs), both in SPC and dementia services:

*I think the lack of knowing each other goes both ways and we really need to work on that—*FG Doctor 4*.*

A minority of focus group and survey participants offered similar insights into how insufficient knowledge among SPC staff pertaining to local dementia-care services might be addressed. The notion of mapping services was pointed to by several participants. For instance, Doctor 2 in the following data extract discusses the concept of a local “dementia care map” as a potentially useful resource for SPC staff.

*… I don’t really know the full extent of dementia services locally—right down to dementia cafés and day centers. A local dementia map would be very helpful and I’m sure a local palliative care map for them would be helpful as well—*FG Doctor 2.

In a similar vein, a survey participant noted that a lack of knowledge surrounding existing local resources equally applies to dementia-care specialists, and suggested that a national level collaborative palliative care approach is much needed among geriatricians and dementia-care specialists to help ensure appropriate care to best meet individual patient and family requirements.

*… ensuring that as specialist geriatric services develop in the community, we clarify what the specialist community palliative services and geriatric services offer, and how the services work together to ensure no duplication or confusion for the patient / family / GP—and that the person is receiving the right care from the service who can best meet their symptoms and other needs. This is a much-needed collaborative national conversation (…)—*Survey Participant (Doctor).

### Educational Areas Related to Providing Clinical Care for PwD

**Figure 2.**
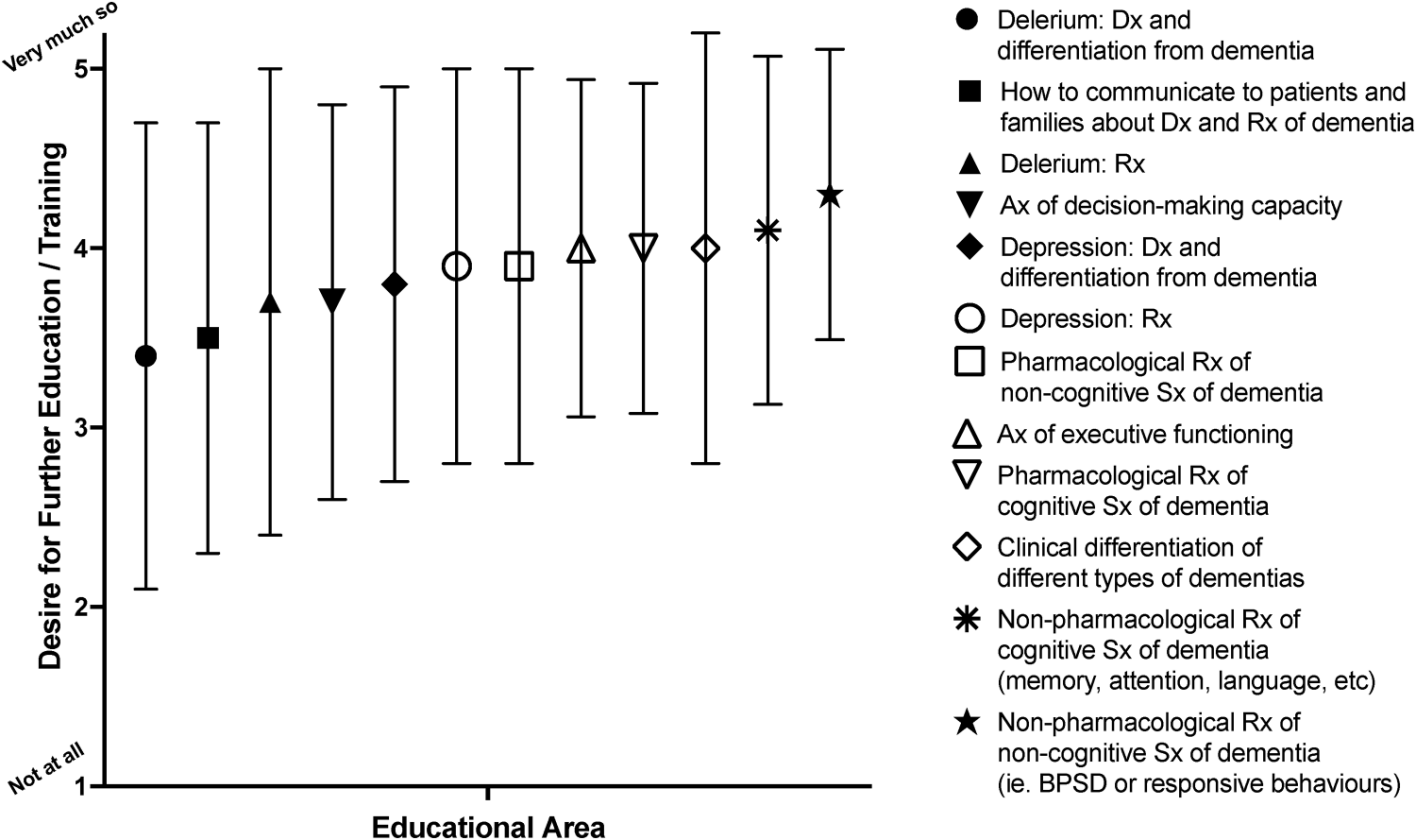
Educational areas related to providing clinical care for PwD; mean (symbol) and standard deviation (line bars) are shown. Abbreviation: PwD, persons with dementia.

Survey participants were asked to rate the extent to which they would like further information and education in several areas related to caring for PwD. There were quite varied responses within most of the individual education topics, but learning needs were relatively balanced across the topics (Figure 2), indicating that all are important to a degree.

SPC ranked their learning needs as highest in the nonpharmacological management of cognitive (4.1 [0.97]) and noncognitive (4.3 [0.81]) symptoms of dementia (alias BPSD), with less variance for these 2 items than others. The pharmacological management of cognitive symptoms (4.0 [0.92]) was also rated highly. Similarly, focus group participants expressed that they were very familiar with drugs used for sedation, and the management of terminal agitation (end-of-life), but unfamiliar with dementia-specific drugs such as acetylcholinesterase inhibitors and memantine. The focus group participants also stated that they had insufficient knowledge concerning the treatment of (nonterminal) noncognitive symptoms of dementia such as agitation, aggression, psychosis, and depression.

Lastly, while no difference was found regarding the identified learning needs between doctors (n = 20) and nurses (n = 44), in addition to nonpharmacological management of noncognitive symptoms of dementia, HSCPs (n = 12) ranked assessment of executive function in PwD to be a major learning need.

The clinical differentiation of dementia subtypes was important to many survey respondents, but with a higher variance (4.0 [1.2]). This topic also arose during the focus group discussion with respect to understanding differences in symptoms, prognostication and disease progression, such as in the following quote:

*… the differentiation of the different dementia syndromes, their prognosis, and what to expect of them. I think when we are not coming from a geriatrics’ perspective, we have a tendency to look at dementia as one illness when it's very much not. It is a spectrum of different illnesses and different patterns of progression. So that would be very useful—to tease that out and have a better knowledge of it—*Doctor 1.

The assessment of executive function was also rated highly by most as an educational topic among survey participants. Interestingly, delirium (a common symptom for PwD) was the least desired educational topic overall, with a large variance between respondents.

### Preferred Modes of Educational Delivery

**Figure 3.**
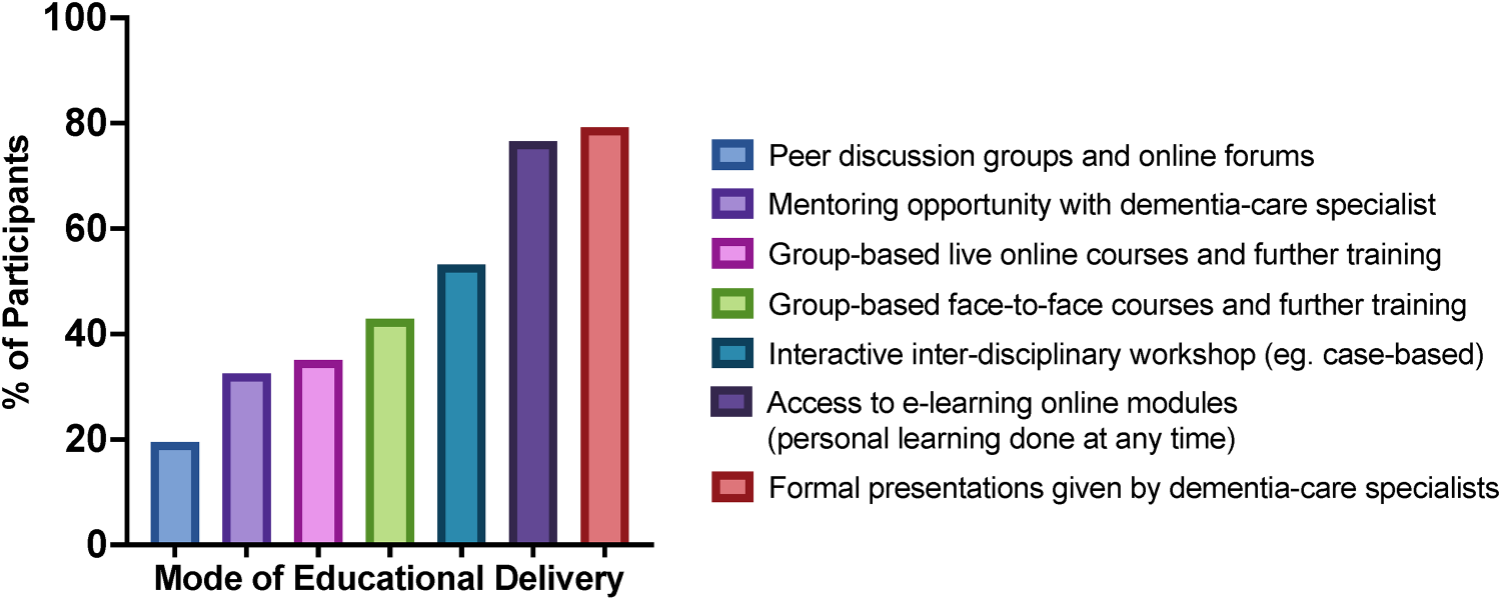
Preferred modes of educational delivery.Abbreviation: BPSD, behavioural and psychological symptoms of dementia.

Survey participants selected their preferred modes of educational delivery (as many as applied) (Figure 3). SPC staff indicated a preference for formal presentations from dementia-care specialists (79.2%) and access to e-learning modules (76.6%), with least interest in peer discussion groups and online forums (19.5%).

Qualitative findings from the survey provided added insight on participants’ perspectives around preferred modes of educational delivery. In particular, there was an emphasis within the open-answer survey questions on collaborative learning with dementia-care specialists. For instance, a survey participant (SPC doctor) commented on case-based collaborative learning being his/her preference, supported by evidence-based didactic teaching with an opportunity for discussion. These findings were reiterated by the focus group participants who likewise addressed the topic of collaborative learning experiences. An example of this is evident in the following extract, in which a doctor expresses that collaborative formal learning between dementia-care specialists and palliative care professionals would be extremely beneficial:

*Perhaps if the Royal College did a joint geriatrics and palliative care master class. I think something like that would be fantastic—*FG Doctor 3.

## Discussion

This is the first study, to our knowledge, to explore the challenges faced and learning needs of SPC staff providing clinical care for PwD. The findings expand upon a literature review by Birch and Draper,^15^ who reported that a lack of knowledge and skills, and difficulty assessing symptoms, is a barrier to delivering effective palliative care for PwD. The findings also confirm the need to upskill SPC staff in dementia care, as part of education and supporting SPC staff with ongoing learning and practice development.^11,14–23^

This study highlights specific challenges for SPC staff in providing clinical care to PwD. Some are similar to those described by Lee et al^27^ for primary care clinicians, such as both groups ranking “managing the needs of PwD” to be an area of specific challenge. However, SPC staff ranked “developing a therapeutic alliance” with PwD to be the least challenging area of care, whereas primary care clinicians identified this to be one of the most challenging.^27^ This may be explained by the fact that palliative care and dementia care share many complementary core principles, including person-centered care, emphasis on a holistic approach, recognition of the importance of nonphysical symptoms, and quality rather than duration of life,^29^ additional to the particular communication skills of SPC staff.

Another key challenge concerned timely access to specialist and community agency support. This could relate to actual service gaps, or due to deficient knowledge of local dementia services availability and best means to interact with these. SPC doctors in this study also perceived that geriatricians were insufficiently aware of local SPC services and access. Similar findings are reported in a national survey in the Netherlands which explored the training requirements of social workers and nurses caring for individuals with intellectual disabilities at end-of-life.^30^ Over 50% of the 181 respondents noted a lack of knowledge pertaining to the availability of consultation facilities externally located, coupled with a need for further training in end-of-life care.^30^

Related to this, previous research by Barber and Murphy^31^ discussed challenges SPC nurses encounter when caring for patients with advanced dementia and reported that knowledge from both specialists in dementia care and palliative care needs to be combined to provide PwD with the highest quality end-of-life care. Thus, mutual service awareness, and understanding each other's roles/access/appropriateness may be a first step toward more integrated or collaborative working to improve the quality of palliative care that PwD receive. A need for integrated services, whereby SPC services work collaboratively with dementia services to develop meaningful and relevant care plans for PwD, is similarly recommended in recent research.^29^ The key components of a palliative care model for dementia was explored via a survey of 112 policymakers, social and healthcare providers, and academics across the Republic of Ireland, Scotland, England, Wales, and Northern Ireland.^29^ An overarching theme was a need for integrated services among palliative and dementia care.^29^ Specific examples included specialists and generalists working collaboratively, such as geriatricians remaining principally involved in care throughout, with the support of SPC when required.^29^

In terms of areas for further education, SPC staff ranked their learning needs as highest in the nonpharmacological management of cognitive and noncognitive symptoms of dementia, clinical differentiation of dementia subtypes, and pharmacological management of cognitive symptoms. These findings mirror those from Lee et al,^27^ where learning needs among primary care clinicians were also highest in the differentiation of dementia subtypes, and nonpharmacological management of symptoms. The need for understanding that “all dementia is not the same” has been highlighted previously, both in terms of prognosis,^32^ and in terms of anticipating and assessing symptoms.^32,33^

With respect to the pharmacological management of cognitive symptoms, SPC staff reported a lack of familiarity around cognitive enhancing medications. SPC staff expressed sufficient familiarity with drugs for managing terminal agitation, but not agitation, aggression, psychosis, and depression outside of end-of-life care. The role of nonpharmacological interventions for noncognitive symptoms of dementia was also a learning need. The EAPC white paper recommended that SPC teams may need additional dementia-care specialist support in managing noncognitive symptoms of dementia.^13^ In Ireland, a Department of Health National Clinical Guideline (NCG) for the appropriate prescribing of psychotropic medication for noncognitive symptoms in PwD has been available since late 2019,^34^ supported by linked guidance on nonpharmacological interventions,^35^ noting that the national implementation of these was delayed by the COVID pandemic. The authors believe that tailored training in this guidance would benefit SPC teams, along with dementia service support for more complex cases.

Participants’ preferences for educational delivery were formal presentations from dementia-care specialists and access to e-learning modules for self-directed, flexible learning. Interestingly, peer discussion groups, group-based training, and interactive case-based workshops were not ranked as highly, although these are often advocated.^36^ This would be congruent with research regarding continuing education in nurse practitioners, where one reported barrier was time pressures and difficulty taking time off work and/or away from patient care.^37^

Lastly, the findings from the focus group, specifically, highlight the beneficial role of SPC in caring for PwD in terms of supporting advance care planning, symptom control, end-of-life care, out-of-hours access, and provision of emotional support. The expertise of SPC in advance care planning is of particular importance, as there is evidence that most PwD do not have discussions around advance care planning with their primary care providers and often caregivers are unaware of their care preferences.^38^ This is despite the known benefits of advance care planning including better end-of-life outcomes (death in preferred place, satisfaction with care, and treatment consistent with wishes).^38,39^ Previous literature has discussed various barriers to discussions around advance care planning in primary care, including lack of time and resources, and the need for further training to support implementation of such discussions.^38,40^ Collaboration between community-based SPC and primary care providers may help to address this, by improving the frequency and quality of discussions around advance care planning with PwD.

This study has several strengths, such as being a national study with a relatively large sample size, from a geographically dispersed sample, including urban and rural practice. It was a mixed-methods study, which allowed for both quantitative and qualitative data to be collected and analyzed. SPC staff from various professions were included, providing a broad understanding of challenges and learning needs across the interprofessional team. Significantly, as outlined above, this was the first study to provide an understanding of challenges and learning needs in SPC staff providing clinical care for PwD. There are, however, some limitations. The survey was originally developed for primary care clinicians and was not validated in an SPC service. Only one focus group was conducted due to the COVID pandemic and resultant increased clinical workloads. Only doctors participated in this focus group, limiting the generalizability to the broader SPC staff population.

A wide range of healthcare professionals such as nurses, doctors, and HSCPs, working directly in SPC, were invited to participate in the study, including the focus group. However, only doctors agreed to participate (and only a small number). It would have been beneficial if SPC staff from multiple backgrounds had participated in the focus group, despite the participating doctors’ high levels of expertise, to gain deeper insight into differing disciplinary needs. Future research could usefully explore these topic areas further.

This study has several implications for future research, and policy development and implementation. Future research involving survey validation among SPC staff would be beneficial. There is also a need for further qualitative research into the learning needs of SPC staff providing clinical care for PwD, with a larger number of focus groups with SPC staff from various professional backgrounds and disciplines. Future research is needed around the development, implementation, and evaluation of educational programs for SPC teams providing clinical care for PwD.

In terms of national policy development and implementation, the study results will be shared with the team currently developing a national model for palliative care in dementia for Ireland.^41^ The results will also be shared with the national implementation group for the NCG on psychotropic medication and nonpharmacological interventions for noncognitive symptoms of dementia, as the study identifies that SPC teams are a key education recipient. There are implications for SPC policy in terms of promotion and facilitation of SPC staff engagement in continuing professional development specifically related to dementia care. There may also need to be more defined guidelines for palliative care for PwD, as well as agreed local pathways of care.

## Conclusion

This was the first study to explore and assess SPC teams’ dementia-care challenges, learning needs, and preferred modes of educational delivery on dementia care. It has demonstrated a strong self-identified need for learning across a broad range of dementia-care topics, including managing the needs of PwD, prognostication, knowledge of local services, nonpharmacological management of noncognitive and cognitive symptoms, differentiation of dementia subtypes, and pharmacological management of cognitive symptoms. In terms of preferred mode of educational delivery, e-learning and presentations from dementia-care specialists were highly valued. The study has also highlighted the need for more integrated dementia service-SPC service working, the first steps to which may be raising mutual awareness of relevant local services and each other's roles and particular skill sets. This may ultimately contribute to improving the quality of palliative care being delivered to PwD.

## Appendix 1: Survey

### SPC Dementia-Care Education Survey

Thank you for considering participating in this survey, which aims to explore specialist palliative care professionals’ education needs related to caring for people with dementia (content, delivery preferences, etc). Ultimately, this may inform the future design and delivery of an educational program for specialist palliative care staff.

Should you choose to participate, you will complete the online survey which follows. The survey involves 3 questions and is expected to take 10-15 minutes to complete.

Participation in this study is completely voluntary. There is no obligation to participate and should you choose to do so you can refuse to answer specific questions or decide not to submit the survey. All information you provide will be confidential and anonymity will be protected throughout the study. IP addresses will not be collected at any point, meaning the data you provide cannot be traced back to you.

You maintain the right to withdraw from the survey at any stage up to the point of data submission. Once submitted, your data will be collated with that of other participants and can no longer be retracted.

The anonymous data will be stored on the University College Cork OneDrive system and subsequently on the UCC server. The data will be stored for 10 years. The information you provide may contribute to research publications and/or conference presentations. The data will also contribute to the primary investigator’s final year medical student research report.

This study has obtained ethical approval from the UCC Social Research Ethics Committee.

If you have any queries about this research, you can contact myself, Sarah Currie, at the following email: 119110093@umail.ucc.ie. Alternatively, you can contact my supervisor, Professor Suzanne Timmons at S.Timmons@ucc.ie.

If you agree to take part in this study, please tick the yes box below, which will bring you to the beginning of the survey.

If you do not agree to take part, simply close this browser. Thank you for your time.

* Required

1. Yes, I am happy to proceed to the survey. *
  Mark only one oval.
   Check here
  Question 1
  The following tasks have been identified as challenges to providing clinical care to people with dementia. In your current practice setting, how challenging do you find the following tasks?
  Please note: 1 = not at all challenging, 3 = neutral, 5 = very challenging. When tasks are not within the scope of your practice or role, please skip that question and move on to the next one.
2. Developing a therapeutic alliance with persons with dementia.
  *Mark only one oval.[Table table2-08258597231180966]*
  **Table table2-08258597231180966:**
  	1	2	3	4	5	
  Not at all challenging						Very challenging
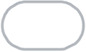
3. Developing a therapeutic alliance with (family) caregivers of persons with dementia.
  *Mark only one oval.[Table table3-08258597231180966]*
  **Table table3-08258597231180966:**
  	1	2	3	4	5	
  Not at all challenging						Very challenging
4. Working collaboratively with other health care professionals involved in dementia care.
  *Mark only one oval.[Table table4-08258597231180966]*
  **Table table4-08258597231180966:**
  	1	2	3	4	5	
  Not at all challenging						Very challenging
5. Working collaboratively with community agencies involved in dementia care.
  *Mark only one oval.[Table table5-08258597231180966]*
  **Table table5-08258597231180966:**
  	1	2	3	4	5	
  Not at all challenging						Very challenging
6. Delivering “bad news” to persons with dementia and their caregivers.
  *Mark only one oval.[Table table6-08258597231180966]*
  **Table table6-08258597231180966:**
  	1	2	3	4	5	
  Not at all challenging						Very challenging
7. Managing the needs of persons with dementia within your current practice setting.
  *Mark only one oval.[Table table7-08258597231180966]*
  **Table table7-08258597231180966:**
  	1	2	3	4	5	
  Not at all challenging						Very challenging
8. Managing the needs of (family) caregivers within your current practice setting.
  *Mark only one oval.[Table table8-08258597231180966]*
  **Table table8-08258597231180966:**
  	1	2	3	4	5	
  Not at all challenging						Very challenging
9. When needed, timely access to community agency support for persons with dementia and their caregivers.
  *Mark only one oval.[Table table9-08258597231180966]*
  **Table table9-08258597231180966:**
  	1	2	3	4	5	
  Not at all challenging						Very challenging
10. When needed, timely access to specialist support for persons with dementia.
  *Mark only one oval.[Table table10-08258597231180966]*
  **Table table10-08258597231180966:**
  	1	2	3	4	5	
  Not at all challenging						Very challenging
11. Establishing and maintaining a positive relationship with persons with dementia.
  *Mark only one oval.[Table table11-08258597231180966]*
  **Table table11-08258597231180966:**
  	1	2	3	4	5	
  Not at all challenging						Very challenging
12. Supporting patients who may not adopt a “sick role”, eg. persons with dementia who have difficulty understanding / accepting their illness.
  *Mark only one oval.[Table table12-08258597231180966]*
  **Table table12-08258597231180966:**
  	1	2	3	4	5	
  Not at all challenging						Very challenging
13. Assessing symptoms (eg. pain, comfort) in persons with dementia.
  *Mark only one oval.[Table table13-08258597231180966]*
  **Table table13-08258597231180966:**
  	1	2	3	4	5	
  Not at all challenging						Very challenging
14. Have you any comments or additions on the above?
    _____________________________________________
    _____________________________________________
    _____________________________________________
    _____________________________________________
    _____________________________________________
  Question 2
  Please rate the extent to which you would like further information and/or education in the following areas.
15. Delirium: Diagnosis and differentiation from dementia
  *Mark only one oval.[Table table14-08258597231180966]*
  **Table table14-08258597231180966:**
  	1	2	3	4	5	
  Not at all						Very much so
16. Delirium: Management
  *Mark only one oval.[Table table15-08258597231180966]*
  **Table table15-08258597231180966:**
  	1	2	3	4	5	
  Not at all						Very much so
17. Depression: Diagnosis and differentiation from dementia
  *Mark only one oval.[Table table16-08258597231180966]*
  **Table table16-08258597231180966:**
  	1	2	3	4	5	
  Not at all						Very much so
18. Depression: Management
  *Mark only one oval.[Table table17-08258597231180966]*
  **Table table17-08258597231180966:**
  	1	2	3	4	5	
  Not at all						Very much so
19. Assessment of executive functioning
  *Mark only one oval.[Table table18-08258597231180966]*
  **Table table18-08258597231180966:**
  	1	2	3	4	5	
  Not at all						Very much so
20. Assessment of decision-making capacity
  *Mark only one oval.[Table table19-08258597231180966]*
  **Table table19-08258597231180966:**
  	1	2	3	4	5	
  Not at all						Very much so
21. Clinical differentiation of the different types of dementias
  *Mark only one oval.[Table table20-08258597231180966]*
  **Table table20-08258597231180966:**
  	1	2	3	4	5	
  Not at all						Very much so
22. Non-pharmacological management of cognitive symptoms of dementia (memory, attention, language, etc)
  *Mark only one oval.[Table table21-08258597231180966]*
  **Table table21-08258597231180966:**
  	1	2	3	4	5	
  Not at all						Very much so
23. Non-pharmacological management of non-cognitive symptoms of dementia (ie. BPSD or responsive behaviours)
  *Mark only one oval.[Table table22-08258597231180966]*
  **Table table22-08258597231180966:**
  	1	2	3	4	5	
  Not at all						Very much so
24. Pharmacological management of cognitive symptoms of dementia
  *Mark only one oval.[Table table23-08258597231180966]*
  **Table table23-08258597231180966:**
  	1	2	3	4	5	
  Not at all						Very much so
25. Pharmacological management of non-cognitive symptoms of dementia
  *Mark only one oval.[Table table24-08258597231180966]*
  **Table table24-08258597231180966:**
  	1	2	3	4	5	
  Not at all						Very much so
26. How to communicate to patients and families about the diagnosis and management of dementia
  *Mark only one oval.[Table table25-08258597231180966]*
  **Table table25-08258597231180966:**
  	1	2	3	4	5	
  Not at all						Very much so
27. Are there other topic areas related to palliative care of persons with dementia that you would like to learn more about?
  _____________________________________________
  _____________________________________________
  _____________________________________________
  _____________________________________________
  _____________________________________________
  Question 3
  Please select your preferred mode of delivery for education. If you would be interested in more than one of the below means of education, please check all that apply.
28. Modes of Education Delivery
  *Check all that apply.*
  Access to e-learning online modules (personal learning done at any time)
  Group-based face-to-face courses and further training
  Group-based live online courses and further training
  Peer discussion groups and online forums
  Interactive inter-disciplinary workshop (eg. case-based)
  Formal presentations given by dementia-care specialists
  Mentoring opportunity with dementia-care specialist
  Other: ________________________________________
29. If you have any further comments on the most appropriate and helpful strategies for receiving training and education related to palliative care of persons with dementia, please comment below.
  _____________________________________________
  _____________________________________________
  _____________________________________________
  _____________________________________________
  _____________________________________________
  Participant Demographics
  Thank you very much for your participation in this survey. If you could take a moment to check the relevant boxes for your details below, that would be appreciated and helpful.
  Thank you.
30. Sex *
  Mark only one oval.
    Male
    Female
    Prefer not to say
    Other: ________________________________________
31. Age *
  Mark only one oval.
    20-34 years old
    35-49 years old
    50+ years old
32. Discipline *
  Mark only one oval.
    Doctor
    Nurse
    Physiotherapist
    Occupational
    Therapist
    Social
    Worker
    Dietitian
    Speech and Language Therapist
    Pastoral Care
    Other
33. If you checked "other" to the above, please indicate your discipline below.
  _____________________________________________
34. Years in Practice *
  Mark only one oval.
    <5 years
    6-15 years
    >15 years
35. General Location in Ireland *
  *Check all that apply.*
    Dublin city or county
    East/North East/Midlands, excluding
    Dublin South/South East/South West
    West/North West
36. Setting(s) of Practice (Please check all that apply) *
  *Check all that apply.*
    Inpatient Hospice
    Inpatient Hospital (Acute)
    Community
    Other
37. If you checked “other” to the above, please indicate your practice setting below.
  _____________________________________________

Thank you and Focus Group Information

Thank you very much for your participation in this survey. Please make sure to click "Submit" below!

We will also be conducting a few focus groups to further explore themes generated from this survey. Focus groups will take place later this year. If you are interested and would like to know further information, please email me, Sarah Currie, at 119110093@umail.ucc.ie.

This content is neither created nor endorsed by Google.

Forms

## Appendix 2: Focus Group Topic Guide

**Welcome**: Welcome and thank you for volunteering to participate in this focus group. You have been asked to participate as your perspective is important. We appreciate your time, thoughts and insight.

**Introduction**: This focus group discussion is designed to explore your perceptions and challenges related to caring for people with dementia (PwD). This may inform the future design and delivery of educational programmes for specialist palliative health care professionals. The focus group discussion will take no longer than 30-40 minutes.

**Anonymity**: As you are aware and consented to, this discussion will be audio-recorded to facilitate data analysis. I would like to assure you that the discussion will be confidential. Once the focus group is completed, the recording will immediately be transferred to an encrypted laptop and cleared from the recording device. The data will then be transcribed by myself, Sarah Currie, and all identifying information will be removed. The audio-recording will be deleted and only the anonymized transcript will remain. You will have 2 weeks after this focus group to retract your data or make clarifications.

Try to answer and comment as accurately and truthfully as possible. Please do not discuss the comments of other participants outside the focus group. If there are any questions that you do not wish to answer, you do not have to do so. However, please try to be as involved and engaged as possible.

**Rules:**

- Only one person should be speaking at a time. If someone is talking and you have something to contribute, please wait until the other person has finished.
- There are no right or wrong responses. Your opinion is valuable.
- When you have something to say, please do so. It is important that I obtain each of your views.
- You do not have to agree with the views of other participants.
- Please let me know if you have any questions at any point.

**Introduction and Initial Question**: First, I would like everyone to introduce themselves by sharing your name, role on the SPC team and the service you are working in.

**Guiding Questions:**

- Are there challenges in providing care to PwD that are different to other palliative care patients?
- What aspects are the same, or easier?
- From your experience, what are the barriers to good dementia care in SPC services?
- From your experience, what are the facilitators of good dementia care?
- What do you think SPC staff need to know about dementia to deliver good dementia care?
- What do you feel SPC staff currently need to learn more about related to caring for PwD?
- What are the most appropriate and helpful strategies for receiving education and training?
- What is your preferred mode of delivery of such education?
- Concluding question: Is there anything else that we should consider when planning education on dementia care for SPC staff?

**Conclusion**: Thank you very much for participating in this discussion. Your thoughts and opinions will be extremely valuable to the study. We hope you have found the discussion to be interesting and engaging. If you are uncomfortable with anything that came up in this focus group, please contact me directly following this focus group at 119110093@umail.ucc.ie. I would like to remind you that all of your comments will be anonymous. Thank you, again, for your time today. We look forward to sharing the results of the study with you in due time.
